# An Innovative Approach in the Baking of Bread with CO_2_ Gas Hydrates as Leavening Agents

**DOI:** 10.3390/foods11223570

**Published:** 2022-11-09

**Authors:** Shubhangi Srivastava, Ann Mary Kollemparembil, Viktoria Zettel, Timo Claßen, Mohammad Mobarak, Bernhard Gatternig, Antonio Delgado, Mario Jekle, Bernd Hitzmann

**Affiliations:** 1 Department of Process Analytics and Grain Science, University of Hohenheim, 70599 Stuttgart, Germany; 2 Institute of Fluid Mechanics (LSTME), Friedrich-Alexander University Erlangen-Nüremberg, 91058 Erlangen, Germany; 3 Process Engineering and Circular Economy, University of Applied Sciences Weihenstephan-Triesdorf, 85354 Triesdorf, Germany; 4 German Engineering Research and Development Center LSTME Busan, Busan 46742, Korea; 5 Department of Plant-Based Foods, Institute of Food Science and Biotechnology, University of Hohenheim, 70599 Stuttgart, Germany

**Keywords:** CO_2_ gas hydrates, bread, leavening, food applications, dough, browning, leavening

## Abstract

Gas (guest) molecules are trapped in hydrogen-bonded water molecules to form gas hydrates (GH), non-stoichiometric solids that resemble ice. High pressure and low temperature are typical conditions for their development, with van der Waals forces joining the host and guest molecules. This article study investigates the application of CO_2_ gas hydrates (CO_2_ GH) as a leavening agent in baking, with particular reference to the production of wheat bread. The main intention of this study is to better understand the complex bread dough formed by CO_2_ GH and its impact on product quality. This may enable the adaptation of CO_2_ GH in baking applications, such as those that can specifically influence wheat bread properties, and so the final bread quality. The present research further examines the comparative evaluation of yeast bread with the GH bread’s impact on bread quality parameters. The amount of GH was varied from 10 to 60%/amount of flour for the GH breads. The GH breads were compared with the standard yeast bread for different quality parameters such as volume, texture, and pore analysis. The results show that the bread with 20% and 40% GH obtained the best results in terms of volume and pore size. Moreover, this article also sheds some light on the future applications of the use of CO_2_ GH as leavening agents in foods. This knowledge could help to create new procedures and criteria for improved GH selection for applications in bread making and other bakery or food products.

## 1. Introduction

Gas hydrates (GH) are formed when water and low molecular weight gases are subjected to low temperature and high pressure conditions. Suitable sized guest molecules are caged in hydrogen-bonded water molecules without chemical reactions to stabilize the structure. The guest molecule may be gaseous or liquid [1]. The most common guest molecules used for the GH are ethane, propane, nitrogen, and carbon dioxide [1,2,3]. The majority (about 85%) of the molecules in a typical GH structure are hydrogen-bonded water molecules that create cages and contain the guest molecules. The water molecules are capable of arranging themselves into cavities (cages) with regular pentagonal and hexagonal faces [4,5]. Since these cages are bigger than crystalline ice holes, the only thing that prevents hydrate cages from collapsing under their attractive forces is the presence of a guest molecule, either in the cage itself or in a significant proportion of the cages around it [6,7], with van der Waals forces joining the host and guest molecules. The process of GH production is considered to be more physical in nature rather than chemical. Additionally, during the production of GH, the guest molecule (CO_2_) freely spins within the cavities of the water molecules [8]. The use of carbon dioxide is approved as E 290 in food bakery units for production. Therefore, the production of CO_2_ GH can be referred to as clean technology [9,10] as the method of production involves only water and CO_2_ (chemical free). In recent years, several food applications of GH have been reported, such as the concentration of fruit juices (apple, pineapple, and orange), and frozen desserts [11,12,13,14,15,16,17,18]. If these technological applications are applied effectively for the CO_2_ GH, then this technology might have the ability to replace existing technologies such as freeze-drying, reverse osmosis, and thermal evaporation for different food products [3].

Carbon dioxide affects the leavening of dough and the bread created from the dough. Numerous efforts have been undertaken to both simplify the process and limit biological CO_2_ generation in doughs or to increase it by adjusting the supplement. Yeast is commonly used in the production of commercial bakery goods such as bread. Yeast plays a crucial role in the process of manufacturing bread by metabolizing carbohydrates and generating carbon dioxide (CO_2_) and ethanol. These substances are created during the dough’s mixing, proofing, and baking [19,20]. Yeast gives bread loaves a slow, consistent rise and greatly enhances their flavor. By enlarging the gas cell diameters in the dough with CO_2_, leavening affects the cellular structure of the dough as well as the bread texture. However, yeast leavening has a lot of disadvantages. First, it takes a lot of time since yeast needs a long time to ferment before it produces enough gas to cause the dough to expand to the desired size. Common industrial techniques for making sponge and straight dough typically take 10–20 min to mix, 1 h to ferment, 10–15 min to prove and form loaves, and then 45–60 min to finish proofing, for a total processing time of 4 to 5 h [21,22]. Another problem that is common in the baking industry is over proofing. In over proofing, generally, the yeast is left to work for too long, so eventually, it becomes exhausted and does not provide enough gas to maintain cells, and the gas bubbles collapse [23]. In other words, during over proofing, the yeast cells typically continue to metabolize the maltose produced by damaged starch hydrolysis [22]. As a result, CO_2_ is produced throughout the entire storage period. High hydration loaves are susceptible to this flaw because their gluten structure is very delicate and deflates easily. A super-dense bottom of bread with large holes on the top is a classic sign of over proofing. Over proofing negatively impacts the rheological and organoleptic characteristics of dough, resulting in low-quality finished loaves [24,25]. Additionally, the dough storage area needs to be large for yeast leavening techniques. Moreover, the energy expended in the manufacture of the baked goods via yeast fermentation lasts for extended periods of time running under strictly controlled environmental conditions (temperature and humidity). Additionally, the fermentation necessitates precise temperature and humidity management, which can be expensive for the bakeries. In addition, biological loosening by yeast is not suitable for all types of dough. For example, components contained in the dough with a high sugar or fat content reduce the activity of the yeast. In these cases, chemical leavening agents are often used in fine baked goods, which produce hydrogen carbonates during the baking process with weak acids and CO_2_. The disadvantage of chemical leavening here is that if the dosage is incorrect then undesirable sensory changes can occur.

Eliminating fermentation and shortening proofing durations would increase productivity in the bread sector. By excluding yeast, it is preferable to manufacture consistent bread products, as with the application of yeast, sometimes due to over proofing or under proofing (fermentation time dependency), the products obtained are not uniform. As an alternative, chemical leavening agents have been employed to leaven the bread by evaporating gas, most often CO_2_ from a chemical source such as sodium bicarbonate or ammonium bicarbonate [26]. These compounds can leaven dough more quickly and easily than yeast and generate bread that resembles bread made with yeast [27,28]. However, the use of chemical leavening agents such as ammonium bicarbonate in bakery products produces acrylamide (cancer producing substances), which poses some major health effects for humans [29,30,31].

In this study, a novel method for producing leavened wheat bread dough that uses new, ecologically friendly, and clean technology is suggested as the CO_2_ gas hydrates (GH). Using CO_2_ GH as the leavening agent instead of yeast, this technology eliminates the expensive and labor-intensive use of yeast and enables the continuous production of ready-to-bake leavened wheat dough. The objective of this study was to develop CO_2_ GH leavened wheat bread comparable to conventional yeast-leavened bread. The CO_2_ GH leavened wheat bread characteristics were studied and compared to those of standard yeast-leavened bread. Bread characteristics such as volume, baking loss, moisture content, and hardness were measured and compared to those of standard yeast bread. Additionally, the total dough making time is reduced when GH is added for bread dough preparation. The total time of dough production is less than one hour when GH are added for bread dough making. This allows for consistent production without the need for the costly storage space requirements for traditional dough fermentation. 

## 2. Materials and Methods

### 2.1. Production of CO_2_ Gas Hydrates (GH) as a Leavening Agent

The CO_2_ GH was produced in a reactor installed at the Department of Process Analytics and Cereal Science, University of Hohenheim, Stuttgart, Germany, in collaboration with installation assistance from the Institute of Fluid Mechanics (LSTME), FAU Erlangen-Nuremberg, Erlangen, Germany [32]. The CO_2_ GH was produced by the addition of 500 mL of distilled water into the reactor vessel connected to the CO_2_ gas cylinder with an optimum pressure of 32 bars and a lower temperature of 1 °C. The CO_2_ GH was produced after a period of 4 h [32], and was stored at −20 °C until further used for the bread baking process.

### 2.2. Bread Making Process Description

The raw materials used to produce standard wheat bread comprise 296.9 g of wheat flour of type 550 purchased from Rettenmeier Mühle GmbH company (Horb am, Germany), 161.8 g of water, 3 g of dry yeast, and 6 g of salt. The amount of water required for the standard bread was determined by the Farinograph-AT (Brabender GmbH & Co. KG). The amount of GH was varied from 10 to 60%/amount of flour for the GH breads. The water content in the GH was measured by weighing 10 g of crushed GH in a weighing balance and again weighing it until all the particles were melted and only water was left after evaporation of the CO_2_ gas. Each 10 g of GH was found to have 1.5 g of CO_2_ and 8.5 g of water. The amount of water present in the GH was subtracted from the total water required for kneading to avoid watery dough. To produce the GH bread, all the dry ingredients were weighed together, and the water was weighed separately. After one minute of mixing all the dry ingredients in the farinograph, water was added along with the respective percentage of GH and kneaded for 3 min. The kneaded dough was proofed at 32 °C for 20 min in a proofing chamber (Wachtel, GmbH, Hilden, Germany). The proofed dough was weighed, divided, and rounded (Brabender GmbH & Co. KG, Duisburg, Germany), followed by shaping and panning. The standard and the GH bread were baked at 220 °C in a baking oven (Wachtel-System STIR, PICCOLO PRO^®^, Wachtel, GmbH, Hilden, Germany) for 30 min with 12 s of initial steaming.

### 2.3. Measurement of Bread Characteristics

The measurements of different bread characteristics were analyzed to compare the standard bread made with yeast with respect to the bread made with GH. Each bread’s characteristics were measured in triplicates. The method followed for measuring the bread characteristics is discussed in the following subsections.

#### 2.3.1. Weight and Baking Loss

The weight of the dough and the weight of the final bread after baking were measured, and baking loss was calculated by Equation (1).

Baking loss (%) = (1 − (Weight of bread/Weight of dough)) × 100(1)

#### 2.3.2. Moisture Analysis

The standard bread and the bread made with GH (10–60%) were checked for moisture content with the use of an infrared moisture analyzer (Kern and Sohn, GmbH, Balingen-Frommern, Germany). 

#### 2.3.3. Volume Analysis

The volume of the standard bread and the bread made with GH (10–60%) was measured for its volume using a volume analyzer (Stable Microsystems, VolScan Profiler 600, Vienna court, United Kingdom). A zero height calibration was performed before the start of the measurement of samples of bread. A three pin stage assembly with a laser focus was used for the volume analysis of bread with a vertical step of 5.0 mm and a rotation speed of 1.0 rps. The specific volume was calculated with the help of Equation (2).

Specific volume (mL/g) = Volume of bread/Weight of bread(2)

#### 2.3.4. Pore Size Analysis

The evaluation of the number and area of the pores was evaluated by a pore scanner (Hp scan jet 5590, Düsseldorf, Germany) connected to an inbuilt software Gebäck analyse version 1.4 with oracle virtual toolbox 6.1. 

#### 2.3.5. Texture Profile Analysis

The bread was sliced into pieces each of 27 mm thickness via a bread slicing machine (ADE Panis 250 model, ADE Germany, GmbH) before the measurement with the texture analyzer. The measurement of hardness (N) for the bread was made with a texture profile analyzer (TA-XT2, Stable Microsystems, Vienna Court, United Kingdom) with a P/36R 36 mm cylindrical probe. 

#### 2.3.6. Bread Pictures Collection

All the bread pictures (standard or GH bread) were captured by a Havox professional photo studio box assembly attached to a Sony alpha ILCE-7 camera. 

## 3. Results and Discussion

### 3.1. Physical Appearance of GH Bread, and Other Characteristics of Bread (Volume Moisture Content, Baking Loss, and Specific Volume)

**Figure 1:**
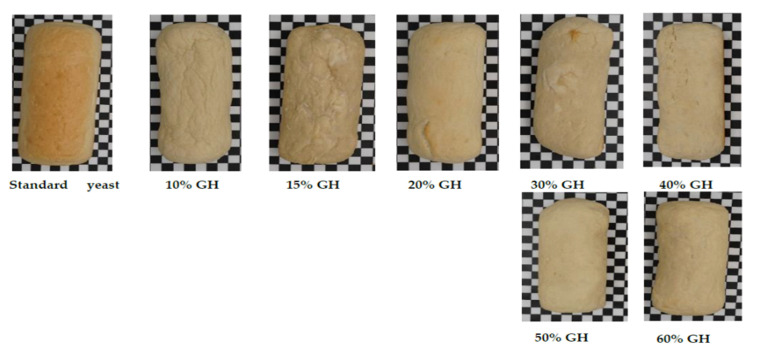
Standard and breads made with different amounts of GH is shown. The pictures of all the breads were taken with a standard black and white checkered box as a background image; the pictures of the bread were taken after resting the bread for a cooling period of 1 h.

Figure 1 shows images of standard yeast bread and GH bread made with different amounts of GH. When comparing the bread with GH (10–60%) with the standard bread, the bread with 20% GH obtained the best appearance, followed by 40%, 50%, and 60%. However, the bread made with 10%, 15%, and 30% GH were not so good in overall appearance. Additionally, it was observed that the bread made with GH had less browning than the standard yeast bread. Yeast affects the color of the bread due to the production of secondary metabolites [33,34] through different metabolic pathways by non-enzymatic chemical reactions such as Maillard and caramelization, which produce brown colored compounds during the baking [35,36,37]. Table 1 shows the characteristics of the standard bread versus the GH bread. The moisture content of all the GH bread was more in comparison to the standard bread. The moisture content of the standard bread was 38.0% while the ones prepared with different percent of GH (10–60%) ranged from 43.5 to 46.9%. However, 50% and 60% GH bread had a lower moisture content than the other percentages of GH. The baking loss for the standard bread was 13.1%. However, in the GH breads, the baking loss (%) values ranged from 6.2 to 10.7%. The maximum baking loss close to the standard bread values was found in 20 (9.8%) and 40 (10.7%) percent GH breads, respectively, indicating a fair range of baking loss with respect to the standard bread. The standard bread volume was around 1193.9 mL while in the case of GH prepared bread, it was almost the half, amounting to a range of 445–559 mL only. The volume characteristics of 20 and 40% GH bread were found to be 635.1 and 559.6 mL, respectively. However, with respect to standard bread, the volume was a little lower (Table 1). Additionally, the specific volumes of 20% (1.4 mL/g) and 40% (1.2 mL/g) GH bread were found to be comparable to the standard bread (2.8 mL/g), which suggests that GH can be used as an alternative to yeast as a leavening agent. However, the volume characteristics of the bread need further reconsideration and have scope for improvement. The volume of each bread and the aerated cell structure of bread are mainly influenced by the addition of yeast metabolism and carbon dioxide production during fermentation [34]. In the absence of yeast, as in the case of GH bread, the gluten matrix is incomplete, as a result of which the released gas is not sufficiently retained in the dough [38]. Thus, the gas holding capacity of the dough is an important characteristic for determining the bread’s quality. The more gas that is trapped in the dough, the smaller the gas cells, and the higher their distribution, which gives a higher specific volume [39,40]. 

Table 1 represents a comparative evaluation of standard bread with the different percentages of GH used for the preparation of bread. Different baking properties were evaluated, such as moisture content, baking loss, volume, and specific volume; the data values of different baking properties of bread are represented in numerical terms along with their standard deviation observed. 

### 3.2. Effect on Hardness and Pores Analysis of GH Bread

**Figure 2:**
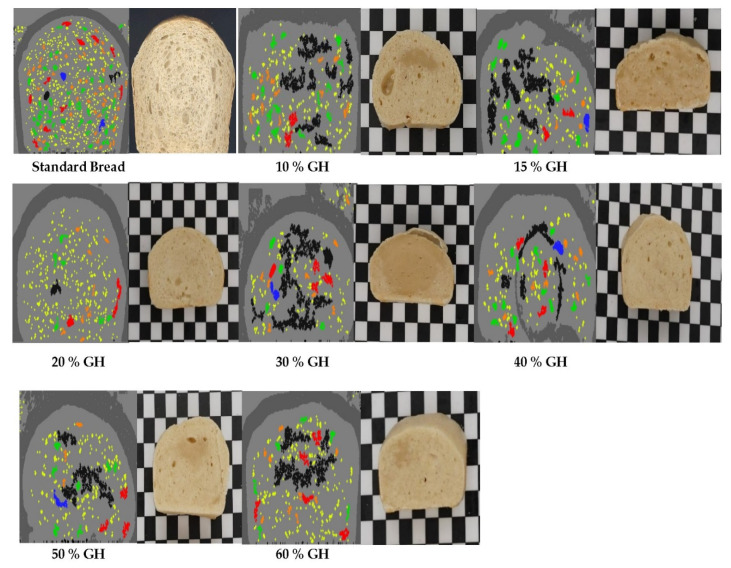
Pore analysis of GH bread versus the standard bread is shown. A comparative evaluation is represented in the pictures, with the left one as the pore size analysis image obtained by software, while right one as the sliced pieces of the bread taken for the pore analysis in each of the respective GH and standard breads.

Table 2 and Figure 2 show the pore analysis of the bread made with GH with respect to the standard bread. The pores were divided as per the mm square size. The small ones were in the range of 0.10–2.00 mm^2^, the medium ones were 3–6 mm^2^, while the larger ones were of 10–11 mm^2^. The number of pores was quite comparable to that of standard bread in the GH prepared bread. The 20% to 60% GH has a higher number of pores in comparison to standard bread. The number of medium and small pores in the GH bread (20–60%) was found to be relatively in proportion with the standard bread (Table 2), but the number of large pores was insignificant in the GH bread. Additionally, the pore analysis depicted via cut sections of the GH bread shown in Figure 2 shows that the 20% and 40% GH breads were better than the other GH % bread. The results thus suggest that gas hydrates actually work to produce some leavening in the bread during the baking process, but still, the bread was not cooked uniformly from the inside as the GH added during the kneading process was not uniformly distributed throughout the dough. Therefore, during baking, some parts of the dough were not leavened properly, resulting in some fractions of uncooked bread. Therefore, this property needs attention and consideration so that the bread with GH is much more comparable to the standard yeast bread that already exists in the bakery units. 

**Figure 3:**
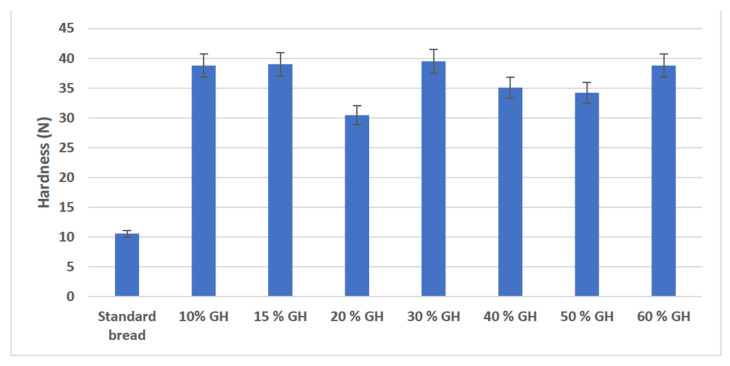
Hardness profile of standard bread vs gas hydrates bread is shown as bar graphs with standard deviation; the x-axis represents the various combinations of GH bread compared with standard bread while the y-axis shows the hardness values of bread in newton.

The hardness profile of standard bread versus gas hydrate bread is shown in Figure 3. When the bread slices were tested for hardness with texture profile analysis, the hardness of all bread made with GH was extremely higher than that of the standard bread. However, the bread with 20% GH had the lowest hardness of all the GH breads prepared, followed by 40% and 50% GH bread. The hardness of the standard bread was near about 10.5 N, while the bread made with GH was quite hard (30.4 N to 39.4 N). In the GH bread, the conversion of starch into simple sugars was affected by the absence of fermentation, which also affected the moisture retention property of the GH bread. The thick crust that was formed in the GH bread may have led to moisture retention inside the crumb, making it harder in texture [35,40]. Another factor that might have led to harder GH bread was the gluten network. In the absence of yeast, the gluten structure was not formed appropriately, as the chemical reactions and biological action of yeast influence the gluten structure, which in turn affects the gas retention capacity of gluten. Thus, yeast, along with gluten, may have a function in the formation of softer bread via the release of moisture during baking [35,41,42]. Another theory that could explain the unbaked portion of the GH bread was that the bread crust formed at the beginning of the baking process might have restricted the gas cells in the unbaked portion of the dough and resulted in increased internal pressure, imposing additional stress on the cell membranes. If the gas cell membranes could not withstand this increase in pressure, the cell membranes would rupture at an early stage and the gas cells would coalesce, resulting in a non-uniform, coarse crumb structure [40,43]. Therefore, this is another criterion that should be taken into consideration when the GH bread recipe improvement is being initiated so that the GH bread made is much softer in texture.

The texture profile analysis of standard bread versus GH bread is shown in Table 3. The texture analysis was classified into six different parameters, such as hardness, springiness, cohesiveness, gumminess, chewiness, and adhesiveness. It was found that the other factors that were mainly affected were the gumminess and chewiness of the GH breads. The chewiness and gumminess values of the standard bread lied in the range of 7.3–7.6 while with the GH breads, the range of 23–28 was observed for the chewiness and gumminess values. The broad change in these values can be attributed to the fact that the hardness of the bread mainly affects the chewiness and gumminess values more than the cohesiveness, and adhesiveness. Moreover, no significant difference was observed in the values of springiness, cohesiveness, and adhesiveness between standard bread and the GH bread (Table 3).

However, this study is the first of its kind and has never been attempted in baking before, so due to the unavailability of research in such an area, it was difficult to relate the work with the ongoing research, but as per the results obtained, it is shown that the recipe for the bread has further scope for improvement. One of the factors by which the recipe can be improved further is with the addition of some promoters while the production of GH is being done or by the addition of some gluten promoting substances in the bread along with the GH as a leavening agent.

Table 2 represents pore size analysis of the standard and GH breads with different amounts of GH; the pores were divided into five different classes (small, somewhat medium, medium, a little bigger, and large) as per the size of the pores in mm^2^; the values of different pore classes are shown along with the standard deviation.

Table 3 represents the texture profile analysis of the standard and GH breads with different amounts of GH; the texture analysis was classified into six different parameters such as hardness, springiness, cohesiveness, gumminess, chewiness, and adhesiveness; the values of the different parameters are shown in the table along with the standard deviation.

## 4. Conclusions

The introduction of CO_2_ GH as a leavening agent for wheat bread in the baking sector is one of the challenges that is addressed in this research. One of the major advantages of CO_2_ GH as a leavening agent would be as an attractive alternative for continuous yeast-free leavened dough production, lack of chemicals, and non-interaction with the sensory properties of the baked product.

When comparing the bread with GH with the standard bread, the bread with 20% and 40% GH obtained the best results in terms of volume and pore size. The baking loss of this bread was also comparable to that of the standard bread. The volume variation observed for the GH bread during baking affected heat and mass transfer owing to pore formation and the consequent variation in properties of the GH bread. Therefore, from the results obtained, 20% and 40% of GH prepared bread have the chance to become better with further changes in the preparation methodology. One of the major challenges faced during the baking of bread with CO_2_ GH was the heterogeneous distribution of the GH during kneading. Additionally, during kneading, cooling down the gluten is a major challenge, as the addition of GH during kneading lowers the temperature of the dough. Furthermore, the GH bread recipe has scope for improvement with the addition of some promoters during the production of CO_2_ GH or by the addition of some gluten promoting substances in the bread along with the GH as a leavening agent.

In recent years, several food applications of GH were reported, such as concentrations of fruit juices (apple, pineapple, and orange), and frozen desserts. However, no literature data were available on the baking aspects of the CO_2_ GH; hence, more sincere and serious efforts are required for the improvement of this technology. However, this research certainly provided some answers to the challenges one can face while working with GH and what measures or methods can be adopted for its further improvement. Moreover, it was found that some of the changes that should be made in the light of the results obtained should be in the direction of modifying the design of the GH reactor, using some additional CO_2_ GH promoting agents (such as food grade amino acids), and modifying the process of bread production with no proofing times. Additionally, perspectives and possible directions in the future application of the use of CO_2_ GH as leavening agents in foods might be in different baking products such as gingerbread, black and white cookies, pound cake, rye bread, etc. (to check what difference does it makes if we change the ingredients of the recipe and the baking method). Furthermore, the production of GH only involves water and CO_2._ No extra chemicals are required for the production. Additionally, with the application of GH, proofing time can be eliminated, saving the cost of the environmental chamber required for yeast fermentation. Moreover, with GH, no such storage of dough is required, and the product can be baked directly. However, the production of GH requires high pressure and low temperature, which can add cost to energy requirements. However, the timing of the baking process becomes more flexible with the use of CO_2_ GH as a leavening agent and can be ideally used in small branch bakeries. Hence, the production of the gas hydrate can be carried out in the bakery on site in a simple manner with a reactor installation (a small area is required), and it can be shifted easily if needed. Therefore, the cost of extended warehousing can be waived.

## Figures and Tables

**Table 1:** The characteristics of standard bread versus GH bread.

	Standard Bread	% of the GH						
		10	15	20	30	40	50	60
Moisture (%)	38.0 ± 2.1	44.0 ± 2.4	46.9 ± 3.4	44.7 ± 1.2	46.3 ± 1.4	45.6 ± 2.7	42.9 ± 2.8	43.5 ± 1.4
Baking loss (%)	13.1 ± 2.4	6.6 ± 3.4	4.4 ± 2.1	9.8 ± 3.7	6.3 ± 3.4	10.7 ± 2.1	7.2 ± 2.5	6.2 ± 2.8
Volume (mL)	1193.9 ± 5.5	518.6 ± 20.0	445.0 ± 31.0	635.1 ± 1.1	445.5 ± 2.3	559.6 ± 28.1	441.4 ± 12.8	497.2 ± 40.2
Specific Volume (mL/g)	2.8 ± 1.2	1.1 ± 0.5	0.9 ± 0.2	1.4 ± 0.2	0.9 ± 0.5	1.2 ± 1.0	0.9 ± 0.1	1.0 ± 0.2

**Table 2:** Pore analysis of standard bread versus the GH bread.

Bread Type	Pore Class (mm^2^)				
	Small (0.10–2.00)	Somewhat Medium (2.01–3.00)	Medium (3.01–6.00)	A Little Bigger (6.00–10.00)	Large (10.00–11.00)
Standard	82.3 ± 0.0%	7.8 ± 1.0%	7.8 ± 1.0%	1.8 ± 0.0%	0.4 ± 0.0%
10% GH	84.5 ± 2.0%	3.7 ± 1.9%	9.0 ± 1.5%	1.5 ± 0.6%	0.7 ± 0.0%
15% GH	81.2 ± 2.0%	8.4 ± 1.4%	9.1 ± 1.9%	1.3 ± 0.7%	0 ± 0.0%
20% GH	93.0 ± 2.4%	3.5 ± 1.3%	2.3 ± 1.0%	1.2 ± 0.2%	0 ± 0.0%
30% GH	86.5 ± 2.8%	4.6 ± 1.7%	4.6 ± 1.3%	3.4 ± 1.2%	0.8 ± 0.0%
40% GH	86.4 ± 2.7%	2.7 ± 1.3%	10.0 ± 1.2%	0.9 ± 0.0%	0 ± 0.0%
50% GH	92.4 ± 2.4%	2.1 ± 1.4%	4.8 ± 1.4%	0.7 ± 0.0%	0 ± 0.0%
60% GH	87.9 ± 2.7%	5.5 ± 1.8%	3.6 ± 1.3%	3.0 ± 1.2%	0 ± 0.0%

**Table 3:** Texture profile analysis of standard bread versus the GH bread.

	Hardness	Springiness	Cohesiveness	Gumminess	Chewiness	Adhesiveness
	N	1	1	N	Nm	g.sec
Std. bread	10.5 ± 2.1	0.9 ± 0.0	0.7 ± 0.0	7.6 ± 2.8	7.3 ± 2.5	−0.01 ± 0.0
10% GH	38.7 ± 3.7	0.8 ± 0.1	0.6 ± 0.1	23.4 ± 2.0	23.6 ± 1.2	−0.02 ± 0.1
15% GH	39.0 ± 3.9	0.8 ± 0.1	0.6 ± 0.1	23.2 ± 3.2	23.9 ± 1.0	−0.05 ± 0.1
20% GH	30.4 ± 3.9	0.9 ± 0.1	0.7 ± 0.0	24.0 ± 2.5	22.3 ± 2.0	−0.08 ± 0.0
30% GH	39.4 ± 3.7	0.8 ± 0.0	0.6 ± 0.1	25.9 ± 2.8	23.2 ± 1.5	−0.06 ± 0.1
40% GH	35.0 ± 3.9	0.8 ± 0.0	0.6 ± 0.1	28.7 ± 2.6	23.5 ± 0.5	−0.04 ± 0.1
50% GH	34.1 ± 4.2	0.9 ± 0.1	0.7 ± 0.0	26.6 ± 3.0	24.1 ± 1.9	−0.01 ± 0.1
60% GH	38.7 ± 5.1	0.8 ± 0.0	0.6 ± 0.1	26.7 ± 2.9	22.7 ± 1.5	−0.09 ± 0.0

## Data Availability

The related data are included in the manuscript.
